# The Homeodomain–Leucine Zipper Subfamily I Contributes to Leaf Age- and Time-Dependent Resistance to Pathogens in *Arabidopsis thaliana*

**DOI:** 10.3390/ijms242216356

**Published:** 2023-11-15

**Authors:** Nami Maeda, Fuko Matsuta, Takaya Noguchi, Ayumu Fujii, Hikaru Ishida, Yudai Kitagawa, Atsushi Ishikawa

**Affiliations:** Department of Bioscience and Biotechnology, Fukui Prefectural University, Fukui 910-1195, Japan

**Keywords:** leaf age, time, HD-Zip I, *Colletotrichum higginsianum*, *Pyricularia oryzae*

## Abstract

In *Arabidopsis thaliana* (Arabidopsis), nonhost resistance (NHR) is influenced by both leaf age and the moment of inoculation. While the circadian clock and photoperiod have been linked to the time-dependent regulation of NHR in Arabidopsis, the mechanism underlying leaf age-dependent NHR remains unclear. In this study, we investigated leaf age-dependent NHR to *Pyricularia oryzae* in Arabidopsis. Our findings revealed that this NHR type is regulated by both miR156-dependent and miR156-independent pathways. To identify the key players, we utilized rice-FOX Arabidopsis lines and identified the rice HD-Zip I *OsHOX6* gene. Notably, *OsHOX6* expression confers robust NHR to *P. oryzae* and *Colletotrichum nymphaeae* in Arabidopsis, with its effect being contingent upon leaf age. Moreover, we explored the role of AtHB7 and AtHB12, the Arabidopsis closest homologues of OsHOX6, by studying mutants and overexpressors in Arabidopsis–*C. higginsianum* interaction. AtHB7 and AtHB12 were found to contribute to both penetration resistance and post-penetration resistance to *C. higginsianum* in a leaf age- and time-dependent manner. These findings highlight the involvement of HD-Zip I AtHB7 and AtHB12, well-known regulators of development and abiotic stress responses, in biotic stress responses in Arabidopsis.

## 1. Introduction

To initiate a plant disease, several factors must interact including the pathogen, host plant, and environmental conditions [1]. It has been observed that the susceptibility of the host plant can vary with the time of day [2]. The circadian clock in plants plays a significant role in regulating plant physiology by integrating environmental cues [3]. Recent studies have shown that the circadian clock can also impact plant responses to biotic stresses [4,5,6]. This biological clock enables plants to anticipate regular environmental changes, such as light and dark cycles, as well as biotic challenges like pathogens.

The susceptibility of host plants to disease also varies with their developmental stage/developmental phenophase [7]. Aging in plants is a complex process that involves various stages such as leaf development, transitions from juvenile to adult plants, and eventual senescence [8]. These stages are genetically programmed and regulated by intricate pathways. As plants age, changes occur in their organ morphology and chemical composition, including alterations in hormone levels. These changes collectively influence how plants perceive and respond to biotic and abiotic stress signals [9]. The stress resilience of a plant organ, such as a leaf, is determined by the integration of age-related developmental factors and stress response pathways. However, the precise molecular and cellular mechanisms underlying this integration remain unclear. Aging in plants can be viewed in two aspects [9,10]. The first is organ aging, which results from the combination of organ differentiation and growth during the plant’s life cycle. Despite the similarity in developmental processes for all organs, differences emerge over time. For example, in *Arabidopsis thaliana* (Arabidopsis), the rosette serves as a developmental axis where leaves of varying ages possess distinct morphologies and biochemistries. Such differences result from a dynamic genetic footprint that changes over time and is influenced by environmental cues. The second aspect of plant aging pertains to the transition from a juvenile to an adult vegetative stage, which then moves into a generative or reproductive phase, activating sexual reproduction. These transitions have significant impacts on the structure and chemistry of existing and developing plant organs and are determined by genetic programming and influenced by environmental factors. One crucial factor in the transition from the juvenile to the adult phase is the *miR156* gene, which is highly expressed in juvenile leaves but decreases as the plant ages [11]. miR156 inhibits the activity of SQUAMOSA PROMOTER BINDING-LIKE (SPL) transcription factors to maintain the vegetative phase and prevent the initiation of flowering [12]. Overexpression of miR156 delays the transition to the adult phase, while its inactivation results in premature flowering.

Resistance to pathogens of a leaf can vary greatly with its age, position, and the age of the plant [7,10,13]. The phenomenon of leaf stage-associated resistance has been examined in various pathosystems, and it has been found to be linked with phytohormones in a way that varies depending on the particular pathosystem [14]. Age-related resistance (ARR) refers to the gain of disease resistance during shoot or organ maturation, and miR156 regulates the timing of ARR associated with the transition from the juvenile to the adult vegetative phase [15]. Despite these findings, our understanding of the molecular mechanisms underlying age-related resistance is still quite limited.

Rice blast is a destructive fungal disease that affects rice and is caused by *Pyricularia oryzae* (syn. *Magnaporthe oryzae*). While rice is a host of *P. oryzae*, most other plants are not. Nonhost resistance (NHR) is the term used to describe the ability of all genotypes of a plant species to provide resistance to all genotypes of a pathogen species. NHR is expressed by every plant towards the majority of potentially pathogenic microbes. Recent studies have identified several rice blast resistance genes in rice, but the mechanisms underlying NHR to *P. oryzae* in nonhost plants are not well understood. To investigate the regulation of NHR to *P. oryzae* in Arabidopsis (a nonhost of *P. oryzae*), we previously identified several genes, including *PENETRATION 2 (PEN2)*, *POWDERY MILDEW RESISTANCE 5 (PMR5)*, and *MILDEW RESISTANCE LOCUS O 2 (MLO2)*, that are involved in NHR [16,17]. *PEN2* encodes an atypical myrosinase that metabolizes indolic glucosinolate (IG) in defense responses [18]. Glucosinolates are secondary metabolites with defensive function in members of the order Brassicales [19]. IGs are derived from tryptophan (Trp), and the first step in IG biosynthesis is catalyzed by CYP79B2 and CYP79B3 [19]. These two P450 monooxygenases convert Trp into indole-3-acetaldoxime (IAOx) [20]. IAOx is a precursor of the IG, camalexin, and indole-3-carboxylic acid derivatives. These indole-type metabolites act in defense responses in Arabidopsis. In fact, *cyp79B2 cyp79B3* mutant plants are highly susceptible to many plant fungal pathogens, including *P. oryzae* [21,22,23,24].

We also found that leaf age and time of inoculation influence NHR in Arabidopsis [25]. Specifically, we discovered that NHR is partially controlled by CIRCADIAN CLOCK ASSOCIATED1 (CCA1) and is thus linked to Arabidopsis’s circadian clock [26]. Additionally, we identified *PMR5* as a candidate gene of direct targets of CCA1 and found that a CCA1-PMR5 module in the epidermis contributes to the establishment of time-of-day-specific NHR to *P. oryzae* in Arabidopsis [27]. However, the role of developmental age in regulating NHR is still unclear.

*C. higginsianum* is a species of *Colletotrichum* that belongs to a main phylogenetic clade within the *C. destructivum* complex. It causes anthracnose disease on a variety of cruciferous plants, including Arabidopsis [28]. Like *P. oryzae*, *Colletotrichum* species produce appressoria-containing melanin in their walls, and their infection mechanism is similar. On Arabidopsis, the hemibiotrophic life cycle of *C. higginsianum* begins with conidia landing on the leaf surface and producing germ tubes that form appressoria to penetrate the leaf surface. Within the breached epidermal cell, the initial narrow hypha from the peg gives rise to a swollen biotrophic hyphae (BH) that enlarges and forms lateral bulbous lobes resembling a haustorium. The fungus establishes itself as a biotroph within 36 h post-infection by forming a multiseptate, multilobed structure that is variable in shape and confined within the initially infected epidermal cells. After 72 h post-infection and subsequent colonization of neighboring cells, there is a switch in both hyphal morphology and the trophic relationship. At the periphery of the lobed BH, outgrowths develop rapidly to produce narrow necrotrophic hyphae (NH). These hyphae radiate from each BH and grow through adjacent cell walls to infect surrounding cells. Narrow NH grow rapidly, and hyphal spread eventually leads to necrotic lesions with the appearance of water-soaked lesions on the surface of the infected host as soon as 84 h post-infection. However, the role of Arabidopsis developmental age in regulating host resistance (HR) to *C. higginsianum* remains unknown.

According to PlantTFDB [29], Arabidopsis and rice have 2296 and 2408 transcription factors (TFs), respectively. These TFs are classified into families based on their DNA binding domain and then divided into subfamilies based on additional structural and functional characteristics. The homeodomain–leucine zipper (HD-Zip) family is unique to plants and is characterized by the presence of a homeodomain linked to a leucine zipper [30]. The HD-Zip family is divided into four subfamilies (I-IV) based on sequence similarity and the intron/exon patterns of the corresponding genes. Members of subfamily I (HD-Zip I) have been found to interact with the pseudo-palindromic sequence CAAT(A/T)ATTG and have been implicated in the plant’s adaptive response to abiotic stress [31]. Their expression is regulated by various external conditions and hormones such as drought, salt, abscisic acid (ABA), ethylene, jasmonic acid, freezing, and aluminum in different tissues and organs [31,32]. In Arabidopsis, the HD-Zip I subfamily comprises 17 members divided into six groups. AtHB7 and AtHB12 belong to the HD-Zip I subfamily in Arabidopsis. Similarly, in rice, OsHOX6, OsHOX22, and OsHOX24, which are the closest homologues to AtHB7 and AtHB12, are upregulated under water-deficit conditions [33,34,35]. *AtHB12* is expressed at higher levels during early Arabidopsis development, while *AtHB7* is expressed during later developmental stages [36]. These two TFs affect each other’s expression, and their regulation is dependent on the plant’s developmental stage, as shown by analyses of gene expression in single and double mutants, and in transgenic plants expressing these TFs. Phenotypic analysis of these plants revealed that AtHB12 induces root elongation and leaf development in young plants under standard growth conditions and seed production in water-stressed plants. In contrast, AtHB7 promotes leaf development, chlorophyll levels, and photosynthesis and reduces stomatal conductance in mature plants. Moreover, AtHB7 delays senescence processes in standard growth conditions [36]. Further, AtHB7 and AtHB12 oppositely regulate aluminum resistance by affecting aluminum accumulation in root cell wall [32]. However, the mechanism by which AtHB7 and AtHB12 regulate development and stress responses is not yet fully understood.

Ichikawa et al. developed the Full-length cDNA OvereXpressing (FOX) hunting system as an alternative to activation tagging in Arabidopsis [37]. In this system, transcriptomes of full-length cDNAs from another plant species are ectopically expressed in Arabidopsis. A rice-FOX Arabidopsis population of 23,000 lines was previously generated by introducing 13,000 full-length rice cDNAs under the control of the cauliflower mosaic virus (CaMV) 35S promoter into Arabidopsis ecotype Columbia [38]. Several screenings have been performed on these lines to date, and genes related to heat stress tolerance [39], salt tolerance [40,41], and disease resistance [42] have been identified.

We discovered that the regulation of NHR to *P. oryzae* involves not only miR156-dependent but also miR156-independent pathways. Using rice-FOX Arabidopsis lines, we identified the *OsHOX6* gene from rice which provides strong NHR to *P. oryzae* and *C. nymphaeae* in the old leaves of rice-FOX Arabidopsis C2-35 plants. The effect of *OsHOX6* expression in Arabidopsis is dependent on the age of the leaves. We also investigated the role of AtHB7 and AtHB12, the closest homologues of OsHOX6 in Arabidopsis, in resistance responses to *C. higginsianum* by studying mutants (*athb7* and *athb12*) and transgenic overexpressors (AT7 and AT12). Our findings reveal that AtHB7 and AtHB12 function in both penetration resistance and post-penetration resistance to *C. higginsianum* in a leaf age- and time-dependent manner.

## 2. Results

### 2.1. Nonhost Resistance to Pyricularia oryzae in Arabidopsis thaliana pen2 35S::miR156a Plants

**Figure 1:**
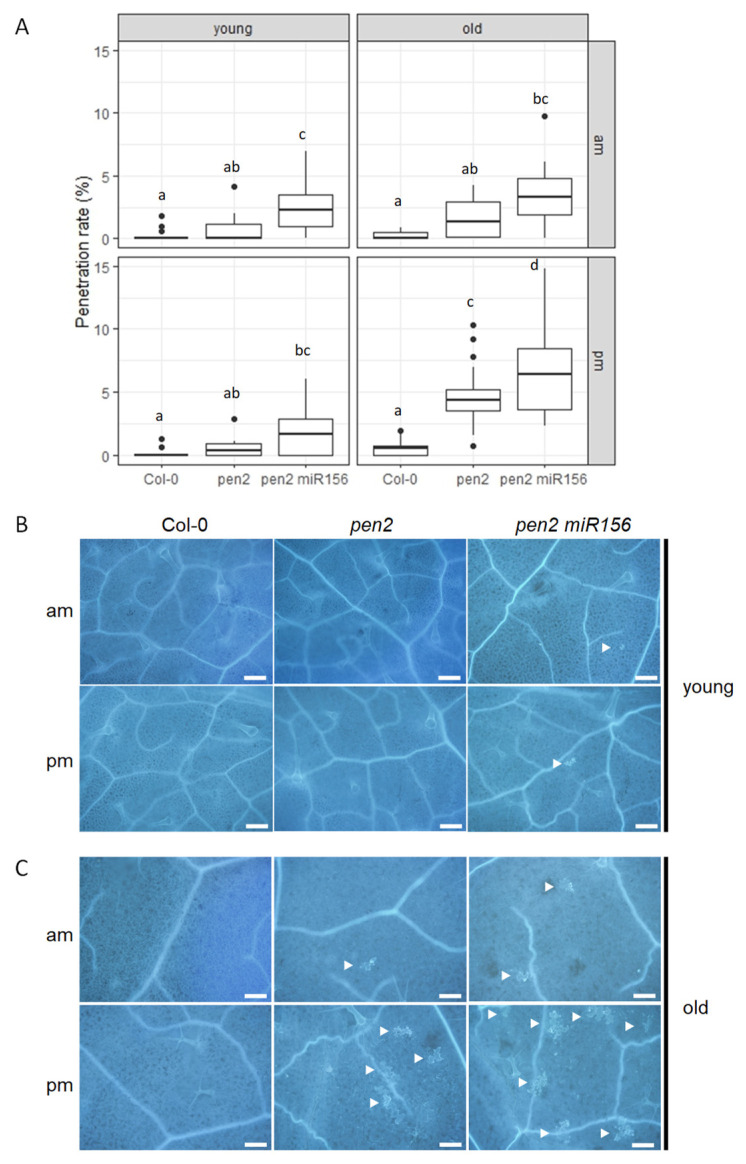
Nonhost resistance to *Pyricularia oryzae* is regulated by miR156-dependent and miR156-independent pathways in *Arabidopsis thaliana* (Arabidopsis). (**A**) Penetration rate of *P. oryzae* into Col-0, *pen2*, and *pen2 35S::miR156a* (*pen2 miR156*) plants at 72 h post-inoculation (hpi) expressed as the percentage of the total number of infection sites. Arabidopsis plants were inoculated at 10:00 a.m. (am) and 5:00 p.m. (pm) on young and old leaves. Values are from three independent experiments, each containing six biological replicates. Significantly different statistical groups of genotypes indicated by the analyses of variance (Tukey’s test; *p* < 0.05) are shown with lowercase letters in each inoculation time. (**B**) Col-0, *pen2*, and *pen2 35S::miR156a* (*pen2 miR156*) plants were inoculated with *P. oryzae* at 10:00 a.m. (am) and 5:00 p.m. (pm) on young leaves. (**C**) Col-0, *pen2*, and *pen2 35S::miR156a* (*pen2 miR156*) plants were inoculated with *P. oryzae* at 10:00 a.m. (am) and 5:00 p.m. (pm) on old leaves. Cell-death-associated autofluorescence at infection sties of Arabidopsis plants at 72 hpi as visualized by fluorescence microscopy. The white arrowhead indicates a penetrated epidermal cell. Bars, 0.2 mm.

In our previous study, we found that *penetration 2* (*pen2*) plants allowed increased penetration into epidermal cells by *P. oryzae*, which suggests the PEN2-dependent nonhost resistance (NHR) to *P. oryzae* in Arabidopsis [16]. We also found that old leaves of *pen2* plants following pm-inoculation showed significantly increased penetration rates compared to wild-type Col-0 plants (Figure 1) [25]. These findings suggest that the regulation of NHR in Arabidopsis *pen2* plants is dependent on both leaf age and the time of day when the plant is inoculated.

We hypothesized that the NHR changes in leaves could be related to phase transitions since the decreased expression of NHR corresponds to the onset of the transition from juvenile to adult leaves in Arabidopsis *pen2* plants (Figure 1) [25]. To investigate this idea, we utilized transgenic Arabidopsis plants overexpressing the miRNA miR156a, which prolongs the expression of juvenile traits [11]. We created *pen2 35S::miR156a* plants and exposed their young and old leaves (leaf numbers 13 and 8, respectively) to *P. oryzae* at two different times, 10:00 a.m. (am-inoculation) and 5:00 p.m. (pm-inoculation), and measured cell penetration (Figure 1). Following am-inoculation, we found that young leaves of *pen2 35S::miR156a* plants showed significantly increased penetration rates compared to *pen2* plants, and the penetration rate in young leaves of *pen2 35S::miR156a* plants was higher than that of old leaves of *pen2* plants (Figure 1). Following pm-inoculation, we found that young leaves of *pen2 35S::miR156a* plants did not show any significant differences compared to *pen2* plants, and the penetration rate in young leaves of *pen2 35S::miR156a* plants was less than that of old leaves of *pen2* plants (Figure 1). These findings suggest that miR156-dependent vegetative phase changes mainly influence NHR following am-inoculation. In contrast, leaf age, rather than vegetative phase change, likely controls NHR following pm-inoculation. Therefore, both miR156-dependent vegetative phase changes and miR156-independent leaf age contribute to the establishment of NHR.

We also found that old leaves of *pen2 35S::miR156a* plants showed a significantly increased penetration rate compared to that of *pen2* plants after pm-inoculation (Figure 1). This result suggests that the miR156-dependent pathway and time of inoculation would synergistically regulate NHR in Arabidopsis.

### 2.2. Nonhost Resistance to Pyricularia oryzae in Rice-FOX Arabidopsis C2-35 Plants

In a previous study, we demonstrated that Arabidopsis *cyp79b2 cyp79b3* plants have reduced NHR to *P. oryzae* compared to *pen2* plants, which suggests that the CYP79B2 CYP79B3-dependent indole-type metabolites act in NHR to *P. oryzae* in Arabidopsis [24]. In order to discover genes related to NHR regulation against *P. oryzae*, particularly in relation to leaf age and time, we developed rice-FOX Arabidopsis lines by introducing rice full-length cDNAs under the control of the cauliflower mosaic virus (CaMV) 35S promoter into Arabidopsis *cyp79b2 cyp79b3* plants.

**Figure 2:**
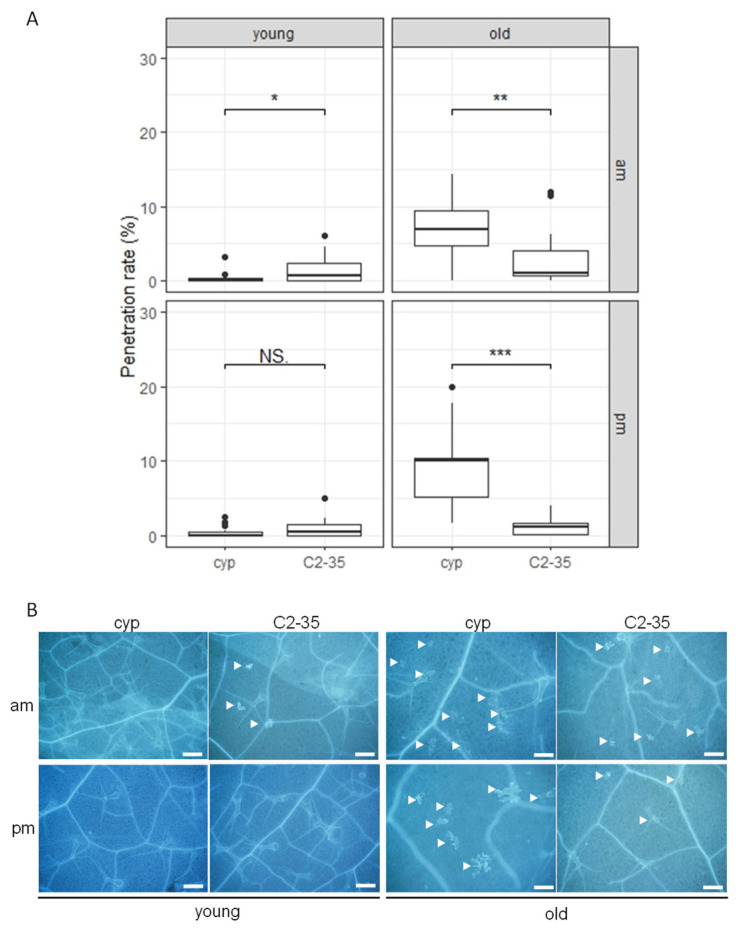
C2-35 plants show increased NHR to *P. oryzae* in old leaves. (**A**) Penetration rate of *P. oryzae* into *cyp79b2 cyp79b3* (cyp) and C2-35 plants at 72 hpi expressed as the percentage of the total number of infection sites. Arabidopsis plants were inoculated at 10:00 a.m. (am) and 5:00 p.m. (pm) on young and old leaves. Values are from three independent experiments, each containing six biological replicates. The Student’s *t*-test was used for statistical analysis; NS, not significant; *, *p* < 0.05; **, *p* < 0.01; ***, *p* < 0.001. (**B**) Arabidopsis *cyp79b2 cyp79b3* (cyp) and C2-35 plants were inoculated with *P. oryzae* at 10:00 a.m. (am) and 5:00 p.m. (pm) on young and old leaves. Cell-death-associated autofluorescence at infection sites of Arabidopsis plants at 72 hpi as visualized by fluorescence microscopy. The white arrowhead indicates a penetrated epidermal cell. Bars, 0.2 mm.

In our study, we first inoculated young rosette leaves of Arabidopsis with *P. oryzae* in the morning (10:00 a.m., am-inoculation) and assessed cell penetration. We screened 500 rice-FOX Arabidopsis *cyp79b2 cyp79b3* lines and identified one line, C2-35, which exhibited decreased NHR to *P. oryzae* compared to the control plants (Figure 2).

To investigate how NHR is regulated in C2-35 plants depending on leaf age and time, we conducted experiments where we inoculated young and old leaves of the plants with *P. oryzae* at 10:00 a.m. (am-inoculation) and 5:00 p.m. (pm-inoculation). Our results show that am-inoculation led to decreased NHR in young leaves but not pm-inoculation (Figure 2). However, both am- and pm-inoculation led to increased NHR in old leaves, which is different from what we observed in young leaves (Figure 2). In contrast to *cyp79b2 cyp79b3* plants, C2-35 plants exhibited a consistently steady penetration rate to *P. oryzae* under various inoculation conditions. These findings suggest that C2-35 plants experience a compromise in leaf age- and time-dependent NHR to *P. oryzae*. We also identified the overexpressed gene in the C2-35 line, which was found to be a full-length rice cDNA AK103160 (Os09g0528200) that encodes rice OsHOX6. These findings indicate that the rice OsHOX6 plays a crucial role in enhancing NHR in old leaves of *cyp79b2 cyp79b3* plants, while not having the same effect on young leaves. In conclusion, our results demonstrate that the regulation of NHR by rice OsHOX6 is dependent on both leaf age and time in Arabidopsis.

### 2.3. Nonhost Resistance to Colletotrichum nymphaeae in Rice-FOX Arabidopsis C2-35 Plants

**Figure 3:**
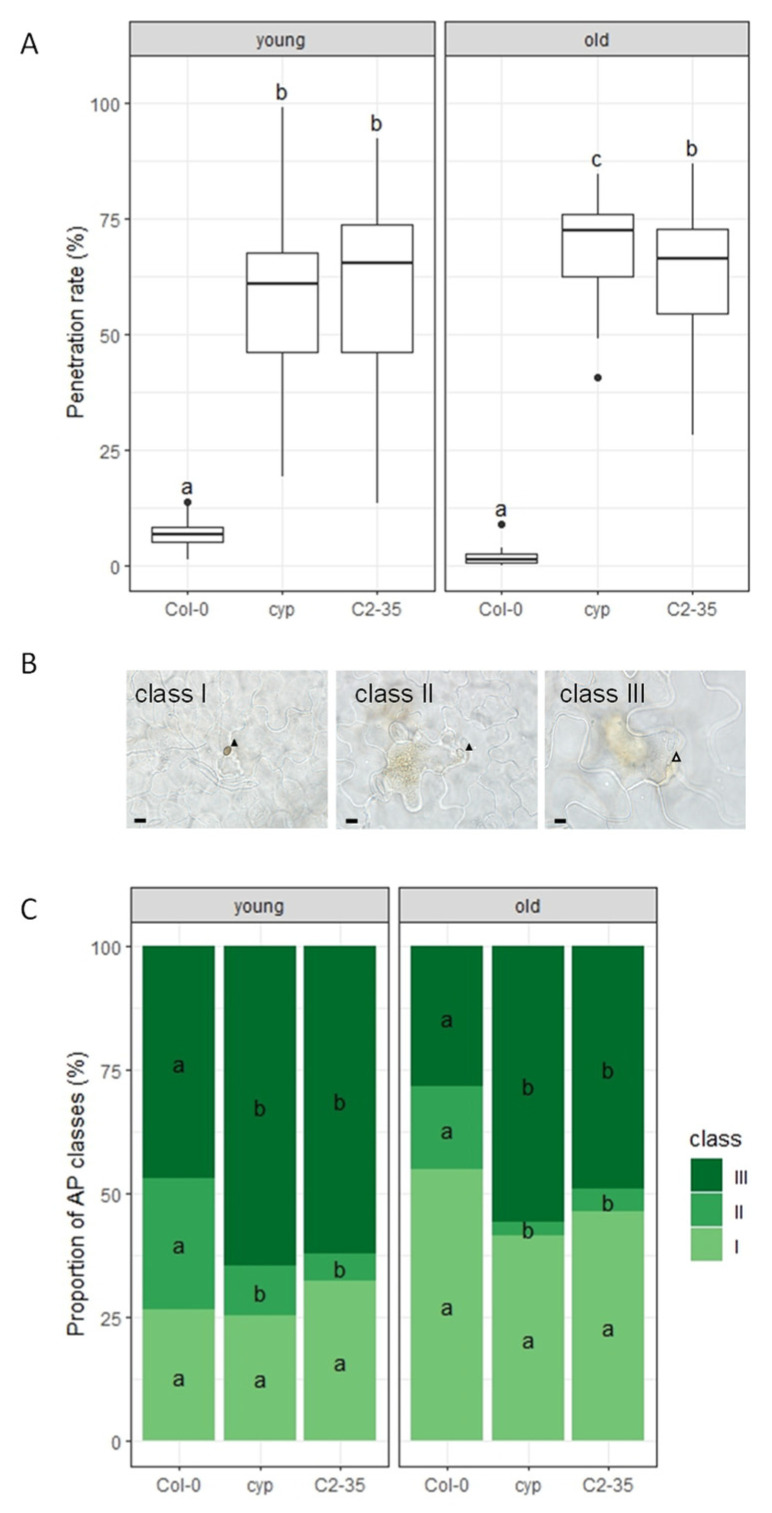
C2-35 plants show increased NHR to *C. nymphaeae* in old leaves. (**A**) Penetration resistance to *C. nymphaeae* in C2-35 plants. Penetration rate of *C. nymphaeae* into Col-0, *cyp79b2 cyp79b3* (cyp), and C2-35 plants at 72 hpi expressed as the percentage of the total number of infection sites. Arabidopsis plants were inoculated at 5:00 p.m. on young and old leaves. Values are from three independent experiments, each containing six biological replicates. Significantly different statistical groups of genotypes indicated by the analyses of variance (Tukey’s test; *p* < 0.05) are shown with lowercase letters. (**B**) Micrographs showing the development of *C. nymphaeae* categorized to different classes (class I–III). Conidial suspensions of *C. nymphaeae* were inoculated on Arabidopsis plants, and penetrated conidia were examined at 72 hpi. Class I, well-melanized appressoria; Class II, slight melanized appressoria or appressoria without detectable pigmentation; Class III, tiny appressoria without detectable pigmentation or penetrated conidia without swollen structures. Bars, 10 μm. Black arrowhead, appressorium; white arrowhead, penetration site of class III. (**C**) Classification of penetrated *C. nymphaeae* appressoria (AP) development. Conidial suspensions of *C. nymphaeae* were inoculated on Col-0, *cyp79b2 cyp79b3* (cyp), and C2-35 plants, and penetrated conidia were examined at 72 hpi. Class I, well-melanized appressoria; Class II, slight melanized appressoria or appressoria without detectable pigmentation; Class III, tiny appressoria without detectable pigmentation or penetrated conidia without swollen structures. Values are expressed as mean from three independent experiments, each containing six biological replicates. Significantly different statistical groups of genotypes indicated by the analyses of variance (Tukey’s test; *p* < 0.05) are shown with lowercase letters.

We inoculated young and old leaves of Arabidopsis plants with *C. nymphaeae* at 5:00 p.m. and quantified cell penetration. This pathogen, isolated from a Japanese flowering cherry, is nonadapted to Arabidopsis [43]. We found that young and old leaves of *cyp79b2 cyp79b3* plants showed significantly increased penetration rates compared to wild-type Col-0 plants (Figure 3A and Figure S1A,B). This result suggests that penetration resistance to *C. nymphaeae* was severely compromised in *cyp79b2 cyp79b3* plants. Further, to assess post-penetration resistance in penetrated epidermal cells, we examined fungal growth in the cells with bright-field microscopy at 72 h post-inoculation (hpi). We measured the severe fungal growth region in the inoculated area of young and old leaves and found that the *cyp79b2 cyp79b3* plants exhibited significantly increased severe fungal growth compared to Col-0 plants (Figure S1D). This result indicates that post-penetration resistance to *C. nymphaeae* was severely compromised in *cyp79b2 cyp79b3* plants compared to Col-0 plants. We also noticed that the severe fungal growth of *C. nymphaeae* in *cyp79b2 cyp79b3* plants damaged infected cells and led to the accumulation of autofluorescent material (Figure S1B).

Then, we tested the effectiveness of rice *OsHOX6* overexpression against nonadapted fungal pathogen *Colletotrichum nymphaeae* by examining the C2-35 lines for NHR. The old leaves of C2-35 lines exhibited increased penetration resistance to *C. nymphaeae*, but not the young leaves (Figure 3A and Figure S1C). This finding indicates that rice OsHOX6 can also provide robust NHR to *cyp79b2 cyp79b3* plants and that the effect is age-dependent on the leaves, similar to NHR to *P. oryzae* in C2-35 plants (Figure 2). In contrast, the growth of infection hyphae in C2-35 plants was comparable to that of *cyp79b2 cyp79b3* plants, suggesting the same level of post-penetration resistance between *cyp79b2 cyp79b3* and C2-35 plants (Figure S1D).

During the experiment, we observed a significant reduction in the incidence of melanized appressoria in the nonadapted *C. nymphaeae*, while host-adapted *C. higginsianum* develops typical melanized appressoria on Arabidopsis. To further understand the infection structure of *C. nymphaeae*, we classified individual germinated spores into three groups. Class I sporelings developed darkly melanized appressoria, while class II sporelings developed an appressorium with slight pigmentation or one without detectable pigmentation. Lastly, class III sporelings produced a germ tube without a recognizable appressorium or developed a small swollen structure at the hyphal tip (Figure 3B). We noticed that the class III hyphal development resembles hyphal tip-based entry (HTE), previously reported in nonadapted *C. gloeosporioides* [44].

We analyzed the proportion of appressoria (AP) classes during *C. nymphaeae* penetration in Arabidopsis. We found that *cyp79b2 cyp79b3* and C2-35 plants exhibited a significant decrease in the incidence of class II sporelings (class II, Figure 3C) and a significant increase in class III sporelings (class III, Figure 3C) compared to wild-type Col-0 plants in young and old leaves (Figure 3C). We could not detect any significant differences between *cyp79b2 cyp79b3* and C2-35 plants (Figure 3C). This result suggests that overexpression of the rice *OsHOX6* gene did not affect the proportion of AP classes during *C. nymphaeae* penetration in Arabidopsis.

### 2.4. Host Resistance to Colletotrichum higginsianum in Rice-FOX Arabidopsis C2-35 Plants

**Figure 4:**
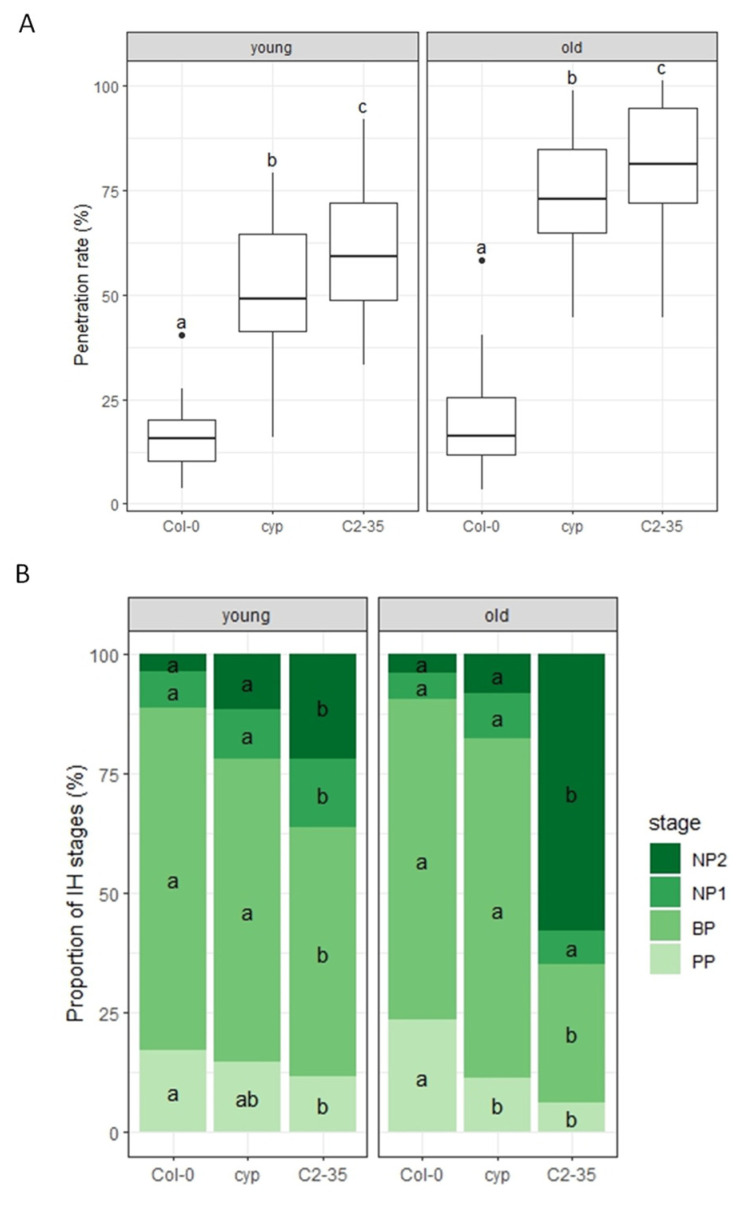
C2-35 plants show decreased host resistance to *C. higginsianum.* (**A**) Penetration resistance to *C. higginsianum* in C2-35 plants. Penetration rate of *C. higginsianum* into Col-0, *cyp79b2 cyp79b3* (cyp), and C2-35 plants at 72 hpi expressed as the percentage of the total number of infection sites. Arabidopsis plants were inoculated at 5:00 p.m. on young and old leaves. Values are from three independent experiments, each containing six biological replicates. Significantly different statistical groups of genotypes indicated by the analyses of variance (Tukey’s test; *p* < 0.05) are shown with lowercase letters. (**B**) Classification of infection hyphae (IH) of *C. higginsianum*. Conidial suspensions of *C. higginsianum* were inoculated on Col-0, *cyp79b2 cyp79b3* (cyp), and C2-35 plants, and IH were examined at 72 hpi. The infection process was classified into four stages: penetration phase (PP), biotrophic phase (BP), necrotrophic phase with NH, which are confined within the initially penetrated epidermal cells (NP1), and necrotrophic phase with NH, which spread into the surrounding cells (NP2). Values are expressed as mean from three independent experiments, each containing six biological replicates. Significantly different statistical groups of genotypes indicated by the analyses of variance (Tukey’s test; *p* < 0.05) are shown with lowercase letters.

To test if overexpression of the rice *OsHOX6* gene could confer resistance to other pathogens, we investigated its effect against the host-adapted *C. higginsianum* in C2-35 plants. We inoculated conidial suspensions of *C. higginsianum* on young and old leaves of Arabidopsis plants at 5:00 p.m. We found that both young and old leaves of *cyp79b2 cyp79b3* plants exhibited a significant decrease in penetration resistance to *C. higginsianum* compared to wild-type Col-0 plants (Figure 4A and Figure S2). Further, we found that both young and old leaves of C2-35 plants exhibited a significant decrease in penetration resistance to *C. higginsianum* compared to *cyp79b2 cyp79b3* plants (Figure 4A and Figure S2). These findings suggest that the overexpression of the rice *OsHOX6* gene can reduce the penetration resistance of young and old leaves of *cyp79b2 cyp79b3* plants against *C. higginsianum*.

We observed that the host-adapted *C. higginsianum* forms specialized infection structures, such as melanized appressoria, penetrating hyphae, biotrophic hyphae, and necrotrophic hyphae, during its infection process on Arabidopsis. Based on this, we divided the process into four stages: the penetration phase (PP), biotrophic phase (BP), necrotrophic phase with NH confined within the initially penetrated epidermal cells (NP1), and necrotrophic phase with NH spreading into the surrounding cells (NP2). Next, we analyzed the proportion of infection stages of penetrated sporelings in Arabidopsis. We could not find any significant differences between Col-0 and *cyp79b2 cyp79b3* plants, except decreased IH in the PP stage in old leaves of *cyp79b2 cyp79b3* plants compared to Col-0 plants (Figure 4B and Figure S2). However, C2-35 plants had significantly higher IH of the NP2 stage in young and old leaves, while significantly lower IH in the BP stage compared to *cyp79b2 cyp79b3* plants (Figure 4B and Figure S2). These results indicate that the overexpression of the rice *OsHOX6* gene significantly reduced post-penetration resistance to *C. higginsianum* in young and old leaves of *cyp79b2 cyp79b3* plants.

### 2.5. Host Resistance to C. higginsianum in Col-0 Plants

The rice *OsHOX6* gene is a member of the HD-Zip I family, with its closest homologues being *AtHB7* (At2g46680) and *AtHB12* (At3g61890) in Arabidopsis. The HD-Zip I family has several members across plant species that regulate development in response to environmental changes. For instance, *AtHB5*, *AtHB6*, *AtHB7*, and *AtHB12* in Arabidopsis are mainly induced by water deficit, salt, and abscisic acid (ABA) [31]. In this study, we found that the rice *OsHOX6* gene plays a role in host resistance (HR) and NHR in rice-FOX Arabidopsis C2-35 plants. This result suggests that AtHB7 and AtHB12 may also regulate resistance responses in Arabidopsis. Furthermore, *AtHB7* and *AtHB12* are induced by various pathogens according to BAR (Bio Analytic Resource for Plant Biology: http://bar.utoronto.ca/, accessed on 11 November 2023), which also implies their involvement in biotic stress responses. However, the precise functions of AtHB7 and AtHB12 in pathogen attack remain poorly understood.

**Figure 5:**
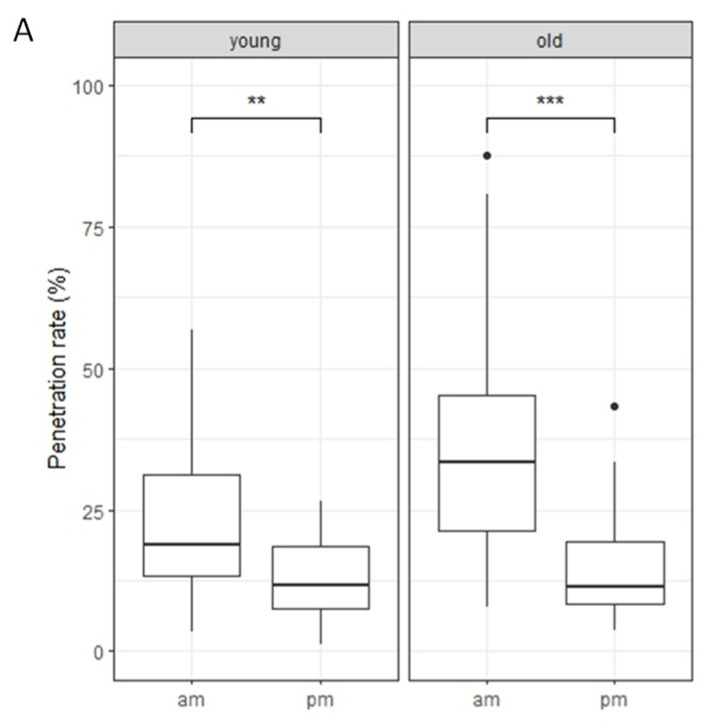

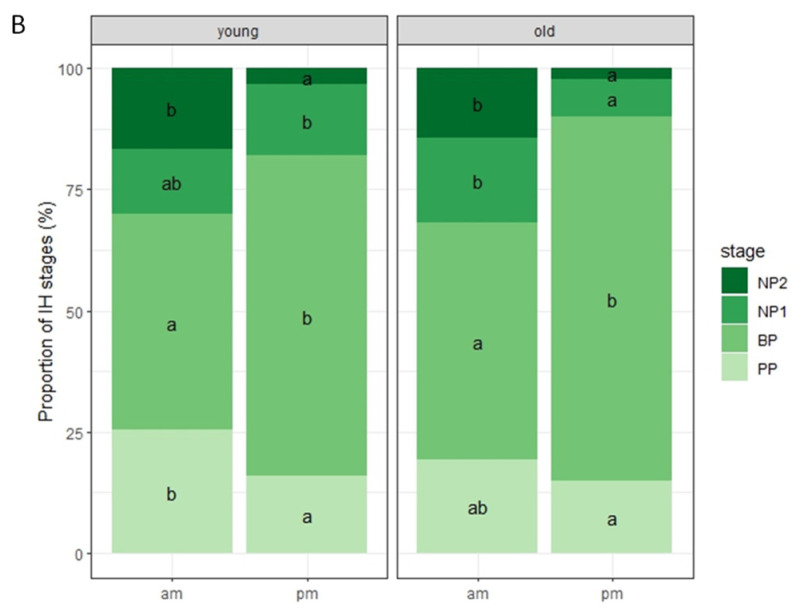
Penetration resistance to *Colletotrichum higginsianum* in Col-0 plants is regulated in a time-dependent manner. (**A**) Penetration resistance to *C. higginsianum* in Col-0 plants. Penetration rate of *C. higginsianum* into Col-0 plants at 72 hpi expressed as the percentage of the total number of infection sites. Arabidopsis plants were inoculated at 10:00 a.m. (am) and 5:00 p.m. (pm) on young and old leaves. Values are from three independent experiments, each containing six biological replicates. The Student’s *t*-test was used for statistical analysis; **, *p* < 0.01; ***, *p* < 0.001. (**B**) Classification of IH of *C. higginsianum*. Conidial suspensions of *C. higginsianum* were inoculated on Arabidopsis, and infection hyphae were examined at 72 hpi. The infection process was classified into four stages: penetration phase (PP), biotrophic phase (BP), necrotrophic phase with NH, which are confined within the initially penetrated epidermal cells (NP1), and necrotrophic phase with NH, which spread into the surrounding cells (NP2). Values are expressed as mean from three independent experiments, each containing six biological replicates. Significantly different statistical groups of genotypes indicated by the analyses of variance (Tukey’s test; *p* < 0.05) are shown with lowercase letters.

We first investigated the interaction between the host-adapted pathogen, *C. higginsianum*, and Arabidopsis Col-0 plants. To do so, we inoculated the plants with conidial suspensions of *C. higginsianum* on young and old leaves of rosettes at two different times, 10:00 a.m. (am-inoculation) and 5:00 p.m. (pm-inoculation). Our findings revealed that Col-0 plants showed a significantly increased penetration rate in young and old leaves after am-inoculation compared to pm-inoculation (Figure 5A). These results suggest that the penetration resistance to *C. higginsianum* in Col-0 plants is mainly regulated in a time-dependent manner.

Following this, we analyzed the proportion of IH stages of penetrated sporelings. Our observations showed that young and old leaves of Col-0 plants had significantly increased IH in the NP2 stage following am-inoculation compared to pm-inoculation (Figure 5B). This indicates that post-penetration resistance to *C. higginsianum* in Col-0 plants is also mainly regulated in a time-dependent manner.

### 2.6. C. higginsianum Growth in Arabidopsis Mutant Plants

To investigate the role of AtHB7 and AtHB12 in Arabidopsis resistance to *C. higginsianum*, mutants (*athb7* and *athb12*) and overexpressors of each gene (AT7 and AT12) were analyzed. Arabidopsis plants were inoculated with *C. higginsianum* conidial suspensions at 10:00 a.m. (am-inoculation) and 5:00 p.m. (pm-inoculation) on young and old leaves of rosettes.

**Figure 6:**
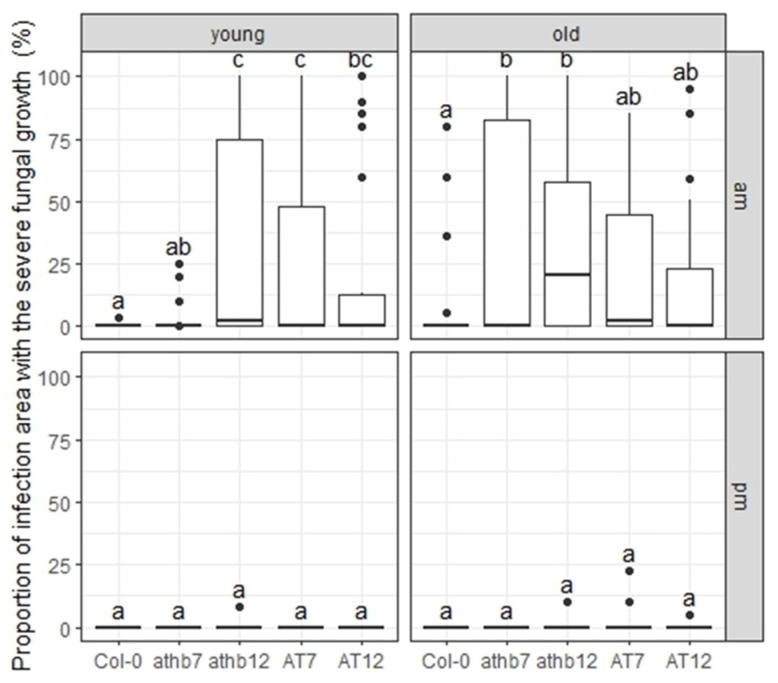
AtHB7 and AtHB12 are involved in time-dependent HR to *C. higginsianum*. Arabidopsis plants, Col-0, *athb7*, *athb12*, AT7, and AT12, were inoculated at 10:00 a.m. (am) and 5:00 p.m. (pm) on young and old leaves with *C. higginsianum*. The proportion of the infection area with the severe fungal growth was measured under microscopy at 72 hpi. Values are from three independent experiments, each containing six biological replicates. Significantly different statistical groups of genotypes indicated by the analyses of variance (Tukey’s test; *p* < 0.05) are shown with lowercase letters.

To assess fungal growth in penetrated epidermal cells, we examined each cell with bright-field microscopy at 72 h post-inoculation (hpi). However, in severe fungal growth regions, it was difficult to distinguish individual epidermal cells due to damage caused by *C. higginsianum* necrotic growth in later phases. Therefore, we first measured the severe fungal growth region in the inoculated area, where penetrated cells were in the NP2 stage, with bright-field microscopy at 72 hpi. After am-inoculation, young leaves of *athb12*, AT7, and AT12 plants exhibited significantly increased severe fungal growth regions compared to Col-0 plants, and old leaves of *athb7* and *athb12* plants also showed significantly increased regions compared to Col-0 plants (Figure 6 and Figure S3). However, no severe fungal growth regions were observed in either young and old leaves following pm-inoculation (Figure 6 and Figure S3). These results suggest that AtHB7 and AtHB12 are involved in time-dependent HR to *C. higginsianum*.

### 2.7. Host Resistance to C. higginsianum in Arabidopsis Mutant Plants

**Figure 7:**
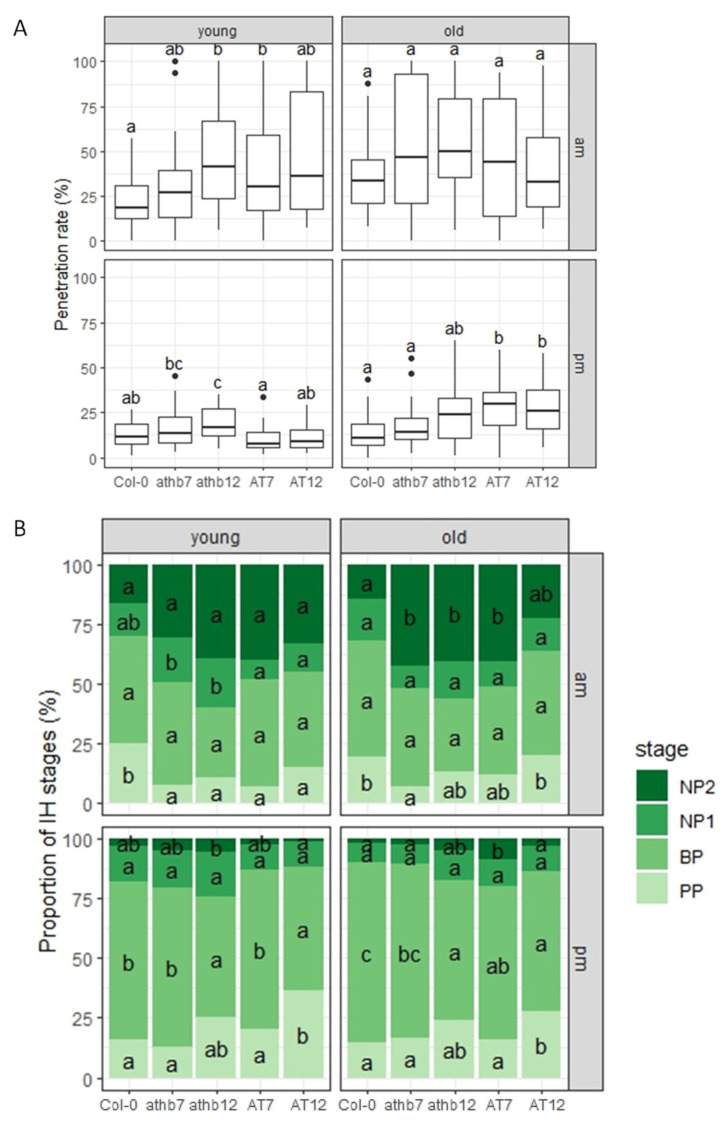
AtHB7 and AtHB12 function in penetration and post-penetration resistance to *C. higginsianum* in a leaf age- and time-dependent manner. (**A**) Penetration rate of *C. higginsianum* into Col-0, *athb7*, *athb12*, AT7, and AT12 plants at 72 hpi expressed as the percentage of the total number of infection sites. Arabidopsis plants were inoculated at 10:00 a.m. (am) and 5:00 p.m. (pm) on young and old leaves. Values are from three independent experiments, each containing six biological replicates. Significantly different statistical groups of genotypes indicated by the analyses of variance (Tukey’s test; *p* < 0.05) are shown with lowercase letters. (**B**) Classification of IH of *C. higginsianum*. Conidial suspensions of *C. higginsianum* were inoculated on Col-0, *athb7*, *athb12*, AT7, and AT12 plants, and infection hyphae were examined at 72 hpi. The infection process was classified into four stages: penetration phase (PP), biotrophic phase (BP), necrotrophic phase with NH, which are confined within the initially penetrated epidermal cells (NP1), and necrotrophic phase with NH, which spread into the surrounding cells (NP2). Values are expressed as mean from three independent experiments, each containing six biological replicates. Significantly different statistical groups of genotypes indicated by the analyses of variance (Tukey’s test; *p* < 0.05) are shown with lowercase letters.

To measure the degree of cell penetration, we conducted an experiment in which we inoculated Arabidopsis plants with conidial suspensions of *C. higginsianum* at 10:00 a.m. (am-inoculation) and 5:00 p.m. (pm-inoculation) on both young and old leaves of the rosettes of Arabidopsis plants. We examined germinated fungal sporelings that had developed appressoria, and we found that the young leaves of *athb12* and AT7 plants showed a significantly increased penetration rate following am-inoculation compared to control Col-0 plants. However, we did not observe any significant differences in the old leaves (Figure 7A and Figure S3). In addition, we found that the young leaves of *athb12* plants and the old leaves of AT7 and AT12 plants showed a significantly increased penetration rate following pm-inoculation compared to control Col-0 plants (Figure 7A and Figure S3). These findings suggest that the *AtHB7* and *AtHB12* genes function in penetration resistance to *C. higginsianum* in a leaf age- and time-dependent manner.

To quantify post-penetration resistance to *C. higginsianum*, we investigated the proportion of different IH stages of penetrated sporelings. We found that following am-inoculation, young leaves of mutants (*athb7* and *athb12*) and overexpressors (AT7 and AT12) showed significantly decreased proportions of the PP stage, while old leaves of mutants (*athb7* and *athb12*) and the overexpressor (AT7) showed significantly increased proportions of the NP2 stage compared to control plants (Figure 7B and Figure S3). These results confirmed the severe fungal growth observed in the previous experiment (Figure 6). Furthermore, following pm-inoculation, significant differences in the proportion of the BP stage of penetrated sporelings were observed in young leaves between mutant plants (*athb12* and AT12) and control plants, and in old leaves between mutant plants (*athb12*, AT7, and AT12) and control plants (Figure 7B and Figure S3). These findings suggest that AtHB7 and AtHB12 play a role in post-penetration resistance to *C. higginsianum* in a leaf age- and time-dependent manner.

## 3. Discussion

In this research, we discovered that leaf age-dependent nonhost resistance (NHR) to *P. oryzae* is not only regulated by miR156-dependent but also miR156-independent pathways. Using rice-FOX *Arabidopsis thaliana* (Arabidopsis) lines, we identified the rice *OsHOX6* gene, which imparts strong NHR to *P. oryzae* and *C. nymphaeae* in the old leaves of Arabidopsis C2-35 plants. The impact of rice *OsHOX6* expression in Arabidopsis is dependent on leaf age. AtHB7 and AtHB12, the Arabidopsis closest homologues of OsHOX6, were then examined in the context of Arabidopsis–*C. higginsianum* interaction by studying mutants (*athb7* and *athb12*) and transgenic overexpressors (AT7 and AT12). Our findings revealed that AtHB7 and AtHB12 play a role in both penetration and post-penetration resistance to *C. higginsianum*, in a manner dependent on leaf age and time.

Age-related changes in immunity are observed in both animals and plants. In plants, age-related resistance (ARR) refers to an increase in disease resistance during the maturation of shoots or organs [10]. This trait is particularly significant during the vegetative phase change, which marks the transition from the juvenile to adult stage. ARR contributes to resistance against multiple pathogens, and recent research by Hu et al. has shown that miR156 plays a crucial role in regulating the timing of ARR [15]. The coordinated development of maturation and the acquisition of disease resistance is achieved through the action of miR156-controlled SPL transcription factors with distinct functions. Specifically, a subset of these factors (SPL2, SPL10, and SPL11) promotes resistance by activating key genes involved in defense signaling [15]. On the other hand, Barens et al. has shown that leaf age controls abscisic acid–salicylic acid cross talk independently of vegetative phase change [45]. Our previous research has revealed that penetration resistance to *P. oryzae* in *pen2* plants is significantly decreased in older leaves following pm-inoculation, as compared to young leaves following am-inoculation [25]. This finding suggests that the circadian clock and developmental age play important roles in NHR to *P. oryzae* in Arabidopsis. In the present study, we have discovered that leaf age-dependent NHR is regulated not only by miR156-dependent pathways but also by miR156-independent pathways (Figure 1).

We have identified the rice *OsHOX6* gene as a key regulator of NHR to *P. oryzae* (Figure 2) and *C. nymphaeae* (Figure 3) in old leaves of rice-FOX Arabidopsis C2-35 plants. Interestingly, we observed that the expression of *OsHOX6* leads to a significant decrease in the HR to *C. higginsianum* in both young and old leaves of C2-35 plants (Figure 4). Our findings suggest that OsHOX6 can function as a regulator of both HR and NHR in Arabidopsis, and its effect on these processes may vary depending on the type of resistance involved. We also investigated the involvement of the closest homologues of OsHOX6 in Arabidopsis, namely, AtHB7 and AtHB12, in leaf age-dependent resistance responses. Previous studies have detected the expression of *AtHB7* and *AtHB12* in meristems, root tips, and flowers and have shown that their expression is strongly upregulated under osmotic or drought stress and when young 14-day-old plants are treated with ABA or NaCl [46,47]. It has been suggested that AtHB7 and AtHB12 act as negative developmental regulators in response to drought [47], and AtHB12 has been assigned a role as a regulator of shoot growth in standard growth conditions [48]. However, the ectopic expression of *AtHB7* in tomato has been shown to confer drought tolerance to this species [49]. Furthermore, loss-of-function *athb7* and *athb12* mutants have been found to activate clade A protein phosphatases 2C (PP2C) genes and repress ABA signaling [50]. In our study, we have demonstrated that AtHB7 and AtHB12 play a role in the plant defense response against *C. higginsianum* in Arabidopsis. Interestingly, the involvement of these TFs is dependent on leaf age and time, as shown in Figure 6 and Figure 7. These findings support the idea that HD-Zip I OsHOX6, AtHB7, and AtHB12 are important in mediating resistance responses in plants. Furthermore, our study highlights the usefulness of rice-FOX Arabidopsis lines in identifying defense-related genes, indicating a shared defense mechanism between monocots and dicots.

The FOX hunting system typically involves the overexpression of full-length cDNA to produce a dominant gain-of-function mutant phenotype. However, in the case of rice-FOX Arabidopsis lines, the resulting plant phenotypes may not accurately reflect the true functions of the overexpressed genes. This is because proteins can be regulated differently in their respective genomic backgrounds due to the difference between amino acid sequences. As a result, overexpressing a foreign gene may lead to different phenotypes than overexpressing the corresponding endogenous gene. Therefore, caution must be exercised when interpreting the phenotypes observed in rice-FOX Arabidopsis lines. To investigate the function of OsHOX6 homologues in Arabidopsis, we examined the role of AtHB7 and AtHB12 in the Arabidopsis–*C. higginsianum* interaction using knockout mutants (*athb7* and *athb12*) and transgenic plants overexpressing these TFs (AT7 and AT12). In this study, we defined knockout mutants as loss-of-function and overexpressors as gain-of-function. Typically, opposite phenotypes are observed between knockout mutants and overexpressors. However, when we examined the function of AtHB7 and AtHB12, we did not observe such opposite phenotypes. Contrary to expectation, both the knockout mutants and overexpressors displayed similar defects in penetration resistance and post-penetration resistance in the Arabidopsis–*C. higginsianum* interaction (Figure 6 and Figure 7). Based on our findings, it appears that the regulation of resistance responses by AtHB7 and AtHB12 is a complex process. The unexpected phenotypes we observed suggest that the expression of these genes involves intricate mechanisms. Recent research by Re et al. has shown that *AtHB12* is highly expressed during the early development of Arabidopsis, while *AtHB7* is expressed more strongly in later developmental stages [36]. This study also demonstrated that these genes have overlapping yet specific roles in various developmental processes. By examining the expression of *AtHB7* and *AtHB12* in single and double mutants, as well as in transgenic plants expressing these genes, the researchers discovered a complex mechanism that depends on the developmental stage of the plant and in which the expression of *AtHB7* and *AtHB12* affects each other [36]. Therefore, to ensure precise function of these transcription factors, it is necessary to fine-tune their expression levels with respect to each other. In knockout mutants and overexpressors, disrupting the expression level of one *AtHB* would also disrupt the expression of the other *AtHB*. This leads to unexpected regulation of *AtHB7* and *AtHB12* and makes it difficult to draw precise conclusions from the analysis of these plants. Nonetheless, our results indicate that AtHB7 and AtHB12 play a role in regulating plant immunity in Arabidopsis. To understand the mechanisms involved, it is important to identify the target genes of AtHB7 and AtHB12 in the context of age- and time-dependent regulation in leaves.

In a previous study, we discovered that Arabidopsis plants exhibited a strong NHR to *P. oryzae* in young leaves after being am-inoculated, while showing a weak NHR in old leaves after being pm-inoculated [25]. However, in our current study, we found that Arabidopsis plants displayed a strong HR to *C. higginsianum* after being pm-inoculated but a weak HR after being am-inoculated. This time-dependent response is different from that seen in the Arabidopsis–*P. oryzae* interaction, despite the similar infection mechanisms used by both pathogens. Our study further revealed that AtHB7 and AtHB12 play a role in resistance against *C. higginsianum* in a time-dependent as well as leaf age-dependent manner, by functioning in both penetration and post-penetration resistance. Additionally, we found that the expression of *AtHB7* and *AtHB12* exhibits a circadian rhythm (CAST-R: https://nagellab.shinyapps.io/CASTR-v1/, accessed on 11 November 2023). These results suggest that the circadian expression of *AtHB7* and *AtHB12* could be responsible for regulating time-dependent HR and NHR in Arabidopsis.

To conclude, the resistance of plants to certain pathogens is affected by the age of leaves and the time of inoculation. In our research, we discovered that the expression of *OsHOX6*, a type of HD-Zip I gene found in rice, can affect NHR against *P. oryzae* and *C. nymphaeae* and HR against *C. higginsianum*, depending on leaf age and time in rice-FOX Arabidopsis C2-35 plants. This implies that the Arabidopsis HD-Zip I genes, *AtHB7* and *AtHB12*, are likely involved in regulating the age- and time-dependent resistance responses in Arabidopsis. Actually, our findings suggest that AtHB7 and AtHB12 are involved in the age- and time-dependent regulation of HR against *C. higginsianum*. Prior studies have indicated that AtHB7 and AtHB12 are associated with the development and responses to abiotic stress in Arabidopsis [36]. Consequently, it would be fascinating to explore how these genes balance the trade-offs between development, abiotic stress, and biotic stress responses in Arabidopsis. To fully comprehend the genetic and mechanistic requirements of AtHB7 and AtHB12 in Arabidopsis, further investigations will be needed. Our study’s insights into the role of AtHB7 and AtHB12 in plant immunity may be used to boost defense responses in plants.

## 4. Materials and Methods

### 4.1. Plant Materials and Growth Conditions

The plants used in this study were Col-0 (wild-type), *pen2* [18], *pen2 35S::miR156a, cyp79b2 cyp79b3* [20], *athb7*, *athb12*, AT7 (*35S::AtHB7*), and AT12 (*35S::AtHB12*) [36], which are all on the Col-0 background. We used *pen2* and *35S::miR156a* [11] plants to generate *pen2 35S::miR156a* plants. *Arabidopsis thaliana* (Arabidopsis) plants were grown on Murashige and Skoog plates in a growth room for three weeks in short-day conditions (9:15 L:D) at 22 °C (in 100 μmol m^−2^s^−1^ fluorescent illumination). Then, the plants were transferred to soil and grown in a growth chamber for 4 weeks, where they continued to grow in short-day conditions (9:15 L:D) at 22 °C (in 100 μmol m^−2^s^−1^ fluorescent illumination).

### 4.2. Fungal Strains and Media

We obtained *Pyricularia oryzae* isolate Hoku 1 (race 007) from H. Koga (Ishikawa Prefectural University). *Colletotrichum higginsianum* (MAFF305635) and *Colletotrichum nymphaeae* (MAFF240037) were obtained from the Ministry of Agriculture, Forestry and Fisheries GenBank, Japan. *P. oryzae* culture was maintained on oatmeal medium at 25 °C in the dark. Cultures of fungal isolates of *Colletotrichum* were maintained on PDA medium at 25 °C in the dark. For inoculation, *P. oryzae* and *C. nymphaeae* were cultured under a 9 h light/15 h dark cycle.

### 4.3. Fungal Inoculation

To measure the penetration rates of fungal pathogens, a conidial suspension of each fungus (*P. oryzae*, 5 × 10^4^ conidia/mL; *Colletrichum*, 1 × 10^5^ conidia/mL) was inoculated onto leaves (young leaf, leaf number 13; old leaf, leaf number 8) of rosettes on Arabidopsis (i.e., leaves numbered from oldest to youngest) in the morning (10:00 a.m., am-inoculation) and the evening (5:00 p.m., pm-inoculation). Inoculated plants were maintained in a growth chamber with saturating humidity in short-day conditions (9 h:15 h light:dark) at 22 °C (in 100 μmol m^−2^s^−1^ fluorescent illumination). Inoculated leaves were harvested at 72 h post-inoculation (hpi).

To quantify cell penetration for *P. oryze* and *C. higginsianum*, we examined germinated fungal sporelings that had developed appressoria (six leaves from six independent plants per experiment and genotype). We evaluated a minimum of 100 appressoria/leaves. We detected successful penetration of fungal pathogens by observing autofluorescence or hyphal elongation at infection sites with fluorescence and bright-field microscopy. Each plant genotype was quantified in three independent experiments.

To quantify cell penetration for *C. nymphaeae*, we examined germinated fungal sporelings. We also examined the appressoria classes that had penetrated into Arabidopsis leaves. We detected successful penetration of *C. nymphaeae* by observing autofluorescence or hyphal elongation at infection sites with fluorescence and bright-field microscopy. Each plant genotype was quantified in three independent experiments.

To quantify severe fungal growth region for *C. nymphaeae* and *C. higginsianum* in Arabidopsis leaves, we examined the inoculated area and measured the severe fungal growth region with bright-field microscopy (six leaves from six independent plants per experiment and genotype). Each plant genotype was quantified in three independent experiments.

### 4.4. Rice-FOX Arabidopsis cyp79b2 cyp79b3 Lines and P. oryzae Screening

The *Agrobacterium* library of rice full-length cDNAs was obtained from RIKEN [38]. Arabidopsis *cyp79b2 cyp79b3* plants were transformed using the *Agrobacterium* library, and the transformed *cyp79b2 cyp79b3* plants expressing rice full-length cDNAs (rice-FOX Arabidopsis *cyp79b2 cyp79b3* lines) were generated. We inoculated the rice-FOX Arabidopsis *cyp79b2 cyp79b3* lines with *P. oryzae* by applying 5 μL droplets (5 × 10^4^ spores/mL) of *P. oryzae* onto young leaves (leaf number 13) of rosettes on Arabidopsis (i.e., leaves numbered from oldest to youngest) in the morning (10:00 a.m.). Then, inoculated plants were maintained in a growth chamber with saturating humidity in short-day conditions (9 h:15 h light:dark) at 22 °C (in 100 μmol m^−2^s^−1^ fluorescent illumination). Inoculated leaves were harvested at 72 hpi. To quantify cell penetration, we examined germinated fungal sporelings that had developed appressoria. We evaluated a minimum of 100 appressoria/leaves. We detected successful penetration of *P. oryzae* by observing autofluorescence or hyphal elongation at infection sites with fluorescence and bright-field microscopy.

We identified the candidate NHR-related lines with a penetration rate different from the rate of control *cyp79b2 cyp79b3* plants from a screen of approximately 500 rice-FOX Arabidopsis *cyp79b2 cyp79b3* lines. Screening of the candidate NHR-related lines was repeated thrice for verification. For further examination, the selected candidate lines were inoculated with *P. oryzae* at 10:00 a.m. (am-inoculation) and 5:00 p.m. (pm-inoculation) on young and old leaves (young leaf, leaf number 13; old leaf, leaf number 8) of rosettes on Arabidopsis. Each plant was quantified in three independent experiments.

## Data Availability

Data are contained within the article and supplementary materials.

## Supplementary Materials

The following supporting information can be downloaded at: https://www.mdpi.com/article/10.3390/ijms242216356/s1.
