# Supplementary material for: The Homeodomain–Leucine Zipper Subfamily I Contributes to Leaf Age- and Time-Dependent Resistance to Pathogens in Arabidopsis thaliana
Source: Int J Mol Sci. 2023 Nov 15;24(22):16356. doi: 10.3390/ijms242216356 (PMC10671646; doi:10.3390/ijms242216356)
Supplement: Supplementary file 1 [file ijms-24-16356-s001.zip › ijms-2714429-supplementary.pdf]

SUPPLEMENTARY MATERIAL

The Homeodomain–Leucine Zipper Subfamily I Contributes to Leaf Age- and Time-Dependent Resistance to Pathogens in *Arabidopsis thaliana*

Nami Maeda, Fuko Matsuta, Takaya Noguchi, Ayumu Fujii, Hikaru Ishida, Yudai Kitagawa, Atsushi Ishikawa

Department of Bioscience and Biotechnology, Fukui Prefectural University, Fukui 910-1195, Japan

***Correspondence**:

Atsushi Ishikawa, ishikawa@fpu.ac.jp

Department of Bioscience and Biotechnology, Fukui Prefectural University, Fukui 910-1195, Japan

A Col-0 young old

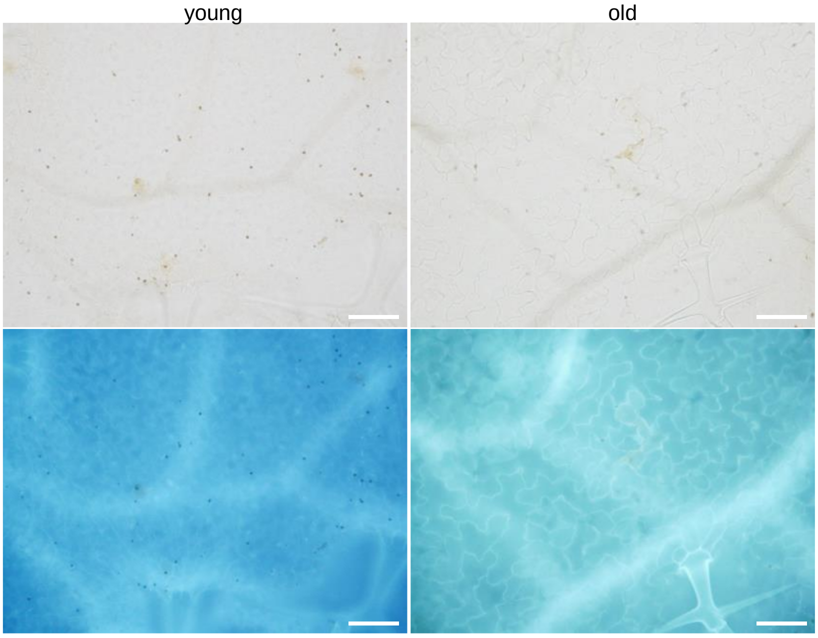

B cyp young old

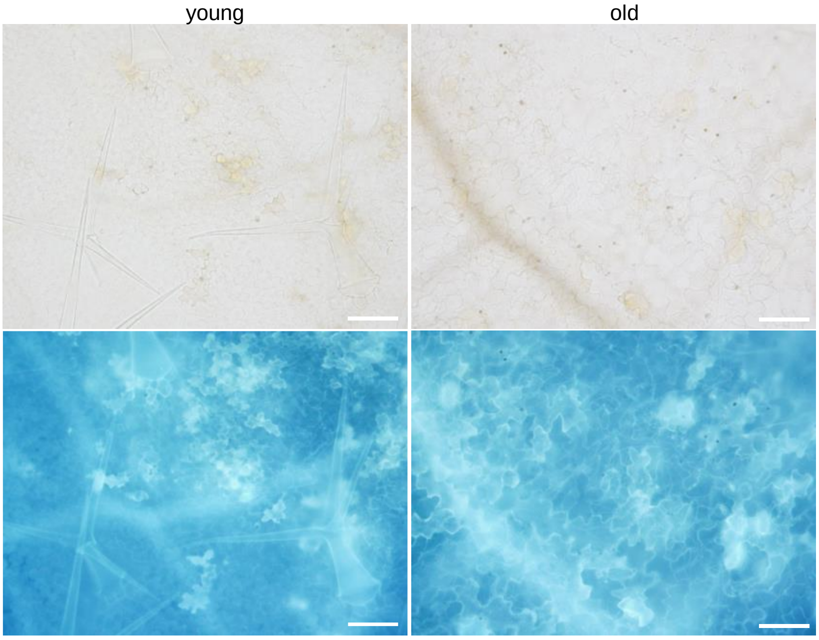

C

C2-35 young old

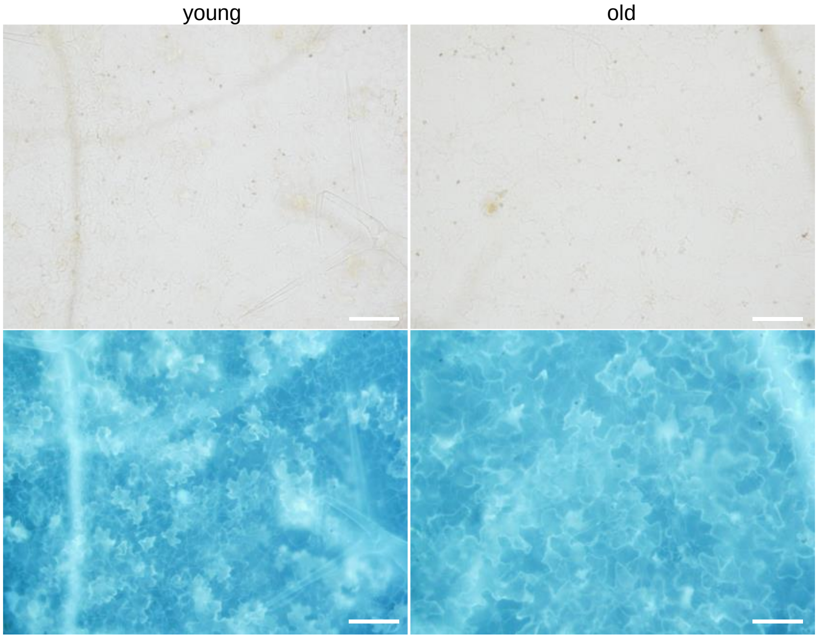

D

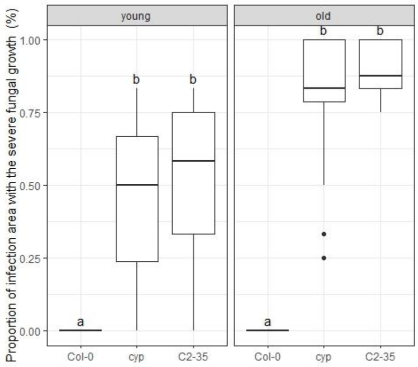

Figure. S1. Nonhost resistance to *Colletotrichum nymphaeae* in C2-35 plants. Arabidopsis Col-0, *cyp79b2 cyp79b3* (cyp) and C2-35 plants were inoculated with *C. nymphaeae* at 5:00 p.m. on young and old leaves. (A-C) Light and fluorescence microscopic views of infection sites of Col-0 (A), *cyp79b2 cyp79b3* (cyp) (B) and C2-35 (C) plants at 72 hpi. Bars, 0.1 mm. (D) Fungal growth in in C2-35 plants. Arabidopsis Col-0, *cyp79b2 cyp79b3* (cyp) and C2-35 plants were inoculated at 5:00 p.m. (pm) on young and old leaves with *C. nymphaeae*. Proportion of infection area with the severe fungal growth were measured under microscopy at 72 hpi. Values are from three independent experiments, each containing six biological replicates. Significantly different statistical groups of genotypes indicated by the analyses of variance (Tukey's test; $p < 0.05$) are shown with lowercase letters.

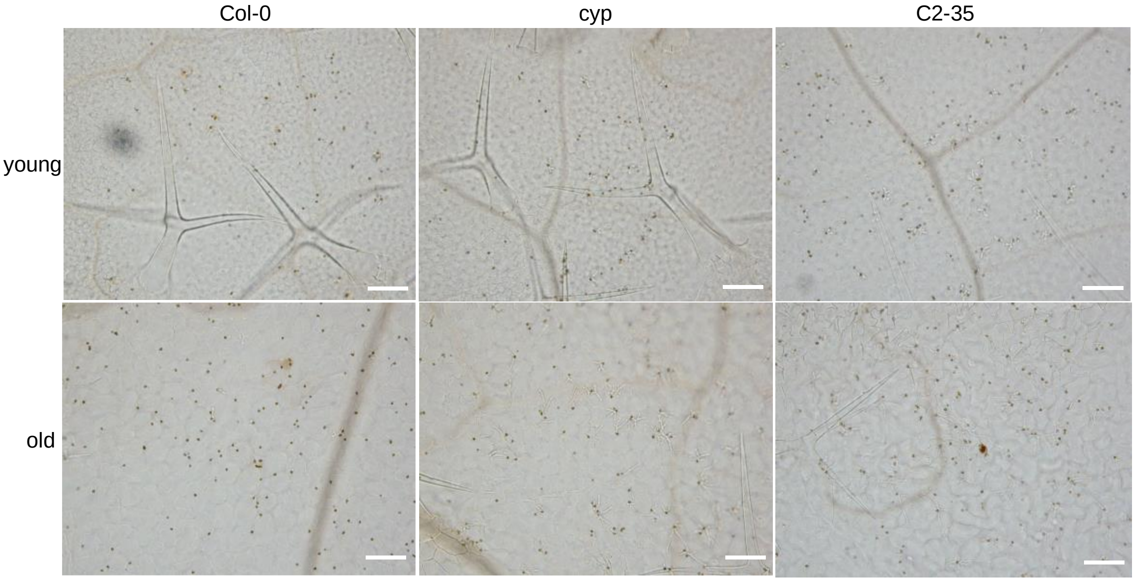

Figure. S2. Host resistance to *Colletotrichum higginsianum* in C2-35 plants. Arabidopsis Col-0, *cyp79b2 cyp79b3* (cyp) and C2-35 plants were inoculated with *C. higginsianum* at 5:00 p.m. on young and old leaves. Light microscopic views of infection sites of Arabidopsis plants at 72 hpi. Bars, 0.1 mm.

A Col-0 young old

am

pm

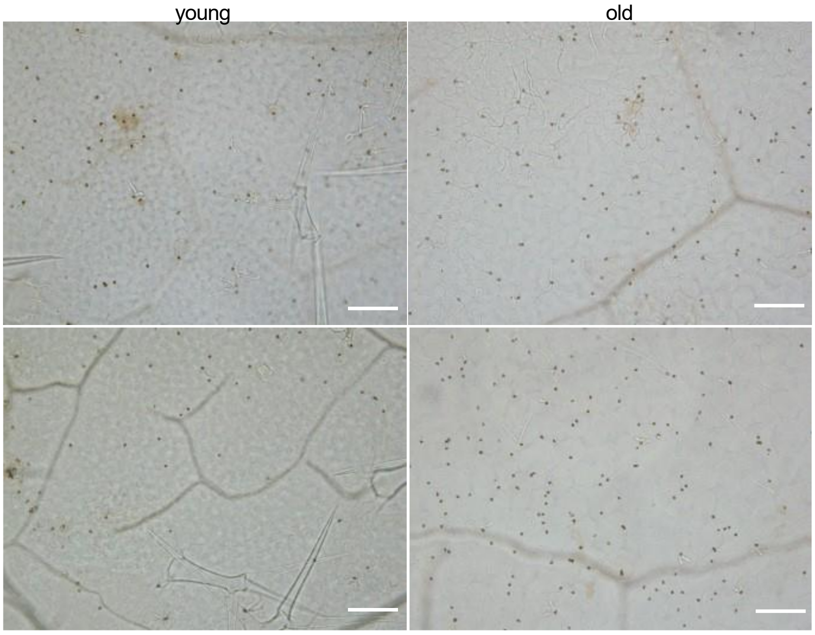

B athb7 young old

am

pm

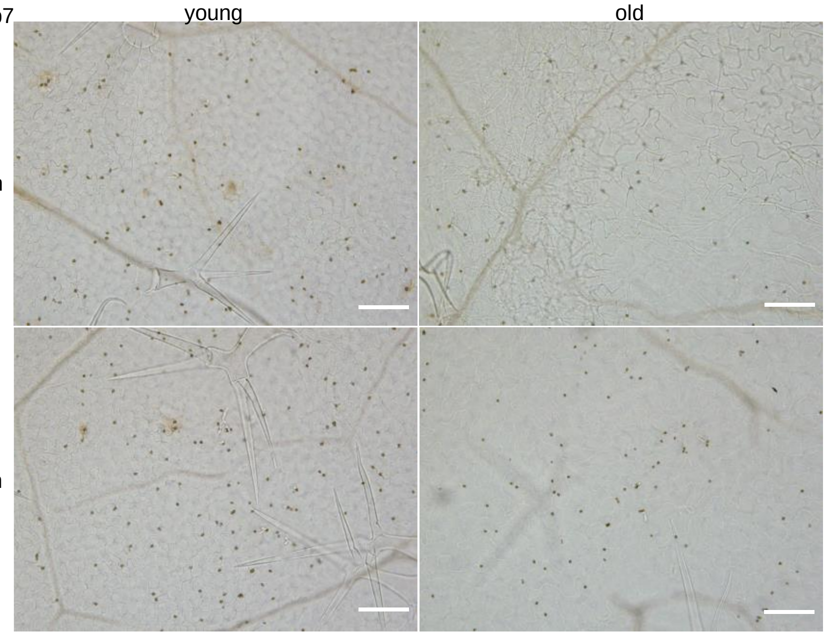

C athb12

young | old

am

pm

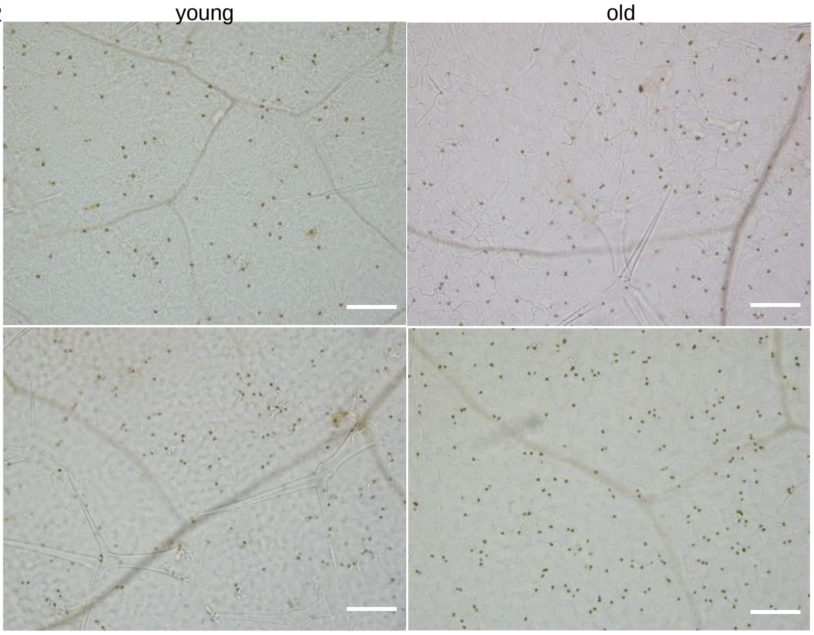

D AT7

young | old

am

pm

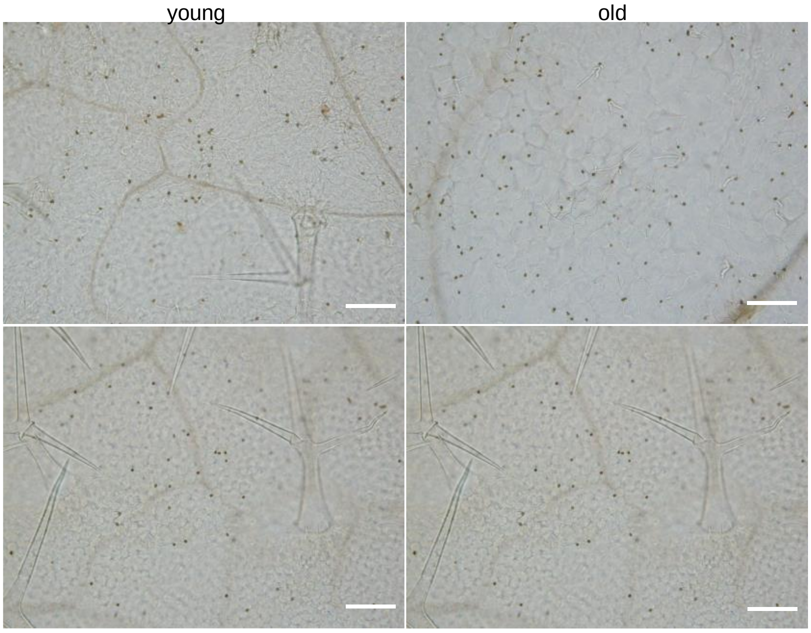

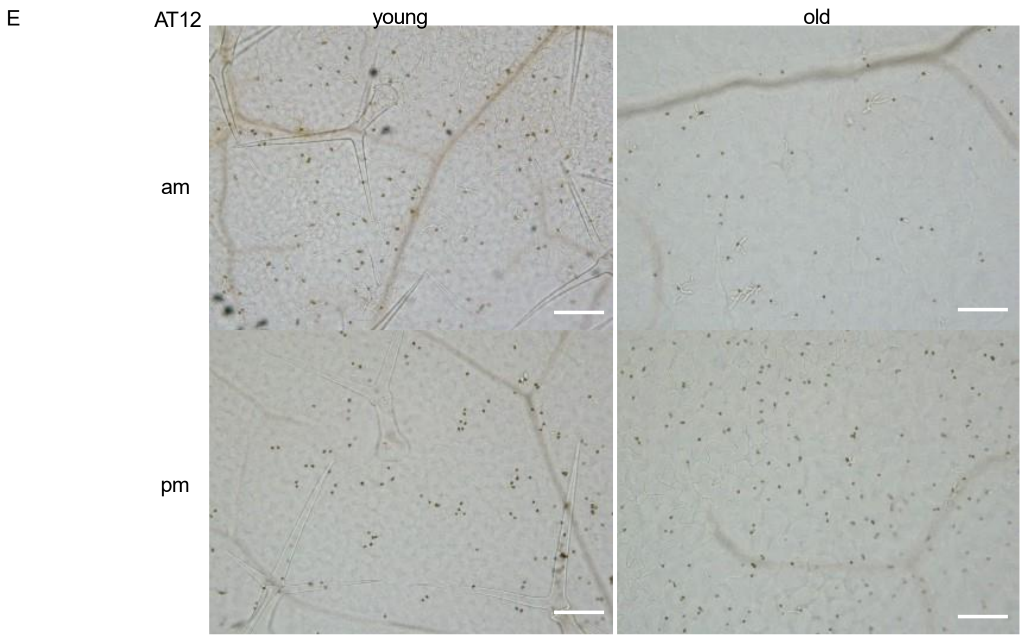

Figure. S3. Host resistance to *Colletotrichum higginsianum* in Arabidopsis mutant plants. Arabidopsis Col-0, *athb7*, *athb12*, AT7 and AT12 plants were inoculated with *C. higginsianum* at 10:00 a.m. (am) and 5:00 p.m. on young and old leaves. (A-E) Light microscopic views of infection sites of Col-0 (A), *athb7* (B), *athb12* (C), AT7 (D) and AT12 (E) plants at 72 hpi. Bars, 0.1 mm.
